# Plasticity results in delayed breeding in a long‐distant migrant seabird

**DOI:** 10.1002/ece3.2777

**Published:** 2017-03-30

**Authors:** F. Stephen Dobson, Peter H. Becker, Coline M. Arnaud, Sandra Bouwhuis, Anne Charmantier

**Affiliations:** ^1^ Centre d'Ecologie Fonctionnelle et EvolutiveUMR 5175 Campus CNRSMontpellier Cedex 5France; ^2^ Department of Biological SciencesAuburn UniversityAuburnALUSA; ^3^ Institute of Avian Research “Vogelwarte Helgoland”WilhelmshavenGermany

**Keywords:** climate, common terns, laying date, micro‐evolution, phenology, phenotypic plasticity

## Abstract

A major question for conservationists and evolutionary biologists is whether natural populations can adapt to rapid environmental change through micro‐evolution or phenotypic plasticity. Making use of 17 years of data from a colony of a long‐distant migratory seabird, the common tern (*Sterna hirundo*), we examined phenotypic plasticity and the evolutionary potential of breeding phenology, a key reproductive trait. We found that laying date was strongly heritable (0.27 ± 0.09) and under significant fecundity selection for earlier laying. Paradoxically, and in contrast to patterns observed in most songbird populations, laying date became delayed over the study period, by about 5 days. The discrepancy between the observed changes and those predicted from selection on laying date was explained by substantial phenotypic plasticity. The plastic response in laying date did not vary significantly among individuals. Exploration of climatic factors showed individual responses to the mean sea surface temperature in Senegal in December prior to breeding: Common terns laid later following warmer winters in Senegal. For each 1°C of warming of the sea surface in Senegal, common terns delayed their laying date in northern Germany by 6.7 days. This suggests that warmer waters provide poorer wintering resources. We therefore found that substantial plastic response to wintering conditions can oppose natural selection, perhaps constraining adaptation.

## 1 Introduction

Seasonal timing of reproduction is an important determinant of reproductive success for many vertebrate species (Verhulst & Nilsson, 2008). In the context of climate change, substantial increases in ambient temperature have repeatedly been related to an advancement of phenology across taxa, including birds (Chambers et al., 2013; Charmantier & Gienapp, 2014; Merilä & Hendry, 2014; Parmesan, 2006; Thackeray et al., 2010). Studies of insectivorous birds, for example, have revealed the need to match the timing of breeding with seasonal peaks in food resources, and that a mismatch occurs when phenology at higher trophic levels advances less than at lower trophic levels (e.g., Both, van Asch, Bijlsma, van den Burg, & Visser, 2009; Charmantier et al., 2008; Donnelly, Caffarra, & O'Neill, 2011; Thackeray et al., 2010; Visser, van Noordwijk, Tinbergen, & Lessells, 1998). Such mistiming can lead to population declines and have dramatic demographic consequences (Both, Bouwhuis, Lessells, & Visser, 2006; Møller, Rubolini, & Lehikoinen, 2008; Saino et al., 2011), although density regulation may (partly) compensate for these effects (Reed, Grotan, Jenouvrier, Saether, & Visser, 2013; Reed, Jenouvrier, & Visser, 2013).

The underlying mechanism of responses to climate changes may be phenotypic plasticity, micro‐evolution, or both (Charmantier & Gienapp, 2014; Gienapp, Leimu, & Merila, 2007; Nussey, Postma, Gienapp, & Visser, 2005; Vedder, Bouwhuis, & Sheldon, 2013; Visser, 2008). Although these two mechanisms can result in similar changes in the mean phenotype in a population, they differ in several fundamental ways. For example, micro‐evolutionary changes and plastic responses can act in opposing directions (e.g., Cooke, Taylor, Francis, & Rockwell, 1990). In addition, the two mechanisms likely differ in the rate of change that they produce, as adaptive evolution is usually a much slower process than phenotypically plastic responses. Determining the limits and interactions of plastic and genetic changes in populations should provide a key to predicting the evolutionary potential and ultimately survival of species, and thus the changes in biodiversity that will be associated with further climate change (Hoffmann & Sgro, 2011).

The evolutionary potential of a trait depends on the amount of additive genetic variation, as well as the genetic correlations between life‐history traits (Blows & Hoffmann, 2005; Falconer & Mackay, 1996; Teplitsky, Robinson, & Merilä, 2014), which may both constrain or facilitate evolutionary change. A constraint will occur, for example, when traits are positively correlated but under opposing selection (Charmantier, Perrins, McCleery, & Sheldon, 2006). Such genetic correlations might be expected between phenological traits involved in the timing of events during the breeding season.

Migratory birds, and especially long‐distance migrants, exhibit a tight annual sequence of interconnected events: fueling at the wintering areas, migration and stopover to refuel en route, arrival at the breeding grounds, and breeding and fueling again for autumn migration (Buehler & Piersma, 2008; Gwinner, 1996). Obviously, birds cannot initiate reproduction before arriving at the breeding ground, finding a partner, settling on a nesting place (Coppack & Both, 2002), and providing or ingesting sufficient food for egg production. The timing of spring migration thus represents a constraint on reproductive timing (Both & Visser, 2001), even more so when migration and reproductive phenology are not only phenotypically but also genetically correlated (Teplitsky, Mouawad, Balbontin, de Lope, & Moller, 2011).

In response to climate change, some migratory species exhibit earlier arrival from migration at the breeding area, while others show no change over time or even delays (reviews in Charmantier & Gienapp, 2014; Gienapp et al., 2007; Gordo, 2007; Lehikoinen & Sparks, 2010). Previous studies have found a genetic basis for migratory behavior (review: Pulido, 2007a; gene identification: Mueller, Pulido, & Kempenaers, 2011) and the timing of migration (Arnaud, Becker, Dobson, & Charmantier, 2013; Pulido, 2007a), such that the investigation of both the phenotypic and genetic correlations between timing of spring migration and the onset of reproduction is essential for understanding micro‐evolutionary adaptability of both phenological traits. Only a few studies of birds have documented micro‐evolutionary adaptability in phenological traits (e.g., Reed, Gienapp, & Visser, 2016), and an analogous study of a small mammalian hibernator demonstrated strong genetic links between the timing of emergence from hibernation and breeding phenology (Lane et al., 2011).

Phenotypic plasticity allows individuals to adjust their phenology to environmental factors that are informative about resource phenology. It is the second mechanism by which natural populations can respond to climate changes and has been more frequently reported than microevolutionary change (general review: Merilä & Hendry, 2014 and further references in the same Special Issue). Although adaptive plasticity has been studied empirically and in theoretical models, some empirical examples suggest that plasticity can also be maladaptive (Langerhans & DeWitt, 2002; Morris & Rogers, 2013). Maladaptive plasticity is theoretically possible in new stressful environments, such as the ones induced by climate change (Ghalambor, McKay, Carroll, & Reznick, 2007).

When exploring the presence of phenotypic plasticity, adaptive or maladaptive, one difficulty is to identify a potential climatic cue. The principal environmental cue identified as a predictor of avian breeding phenology is photoperiod (Dawson, 2008; Lambrechts & Perret, 2000); but in the face of unpredictable environmental variation, the use of other cues, such as temperature, is possible (Visser, 2008). However, as for global climatic factors such as North Atlantic Oscillation (NAO) Index, temperature can only be weakly correlated with the underlying causal factor (e.g., food resources), leading to an underestimate of phenotypic plasticity (reviewed by Charmantier & Gienapp, 2014). Long‐distance migrants are species for which identifying the environmental factors affecting phenology is particularly difficult (Gremillet & Charmantier, 2010; Szostek, Bouwhuis, & Becker, 2015), and the comparative extent to which climatic variables in the wintering area, along the migration route, and at the breeding grounds influence avian migration and subsequent reproductive phenology is unclear.

**Figure 1:**
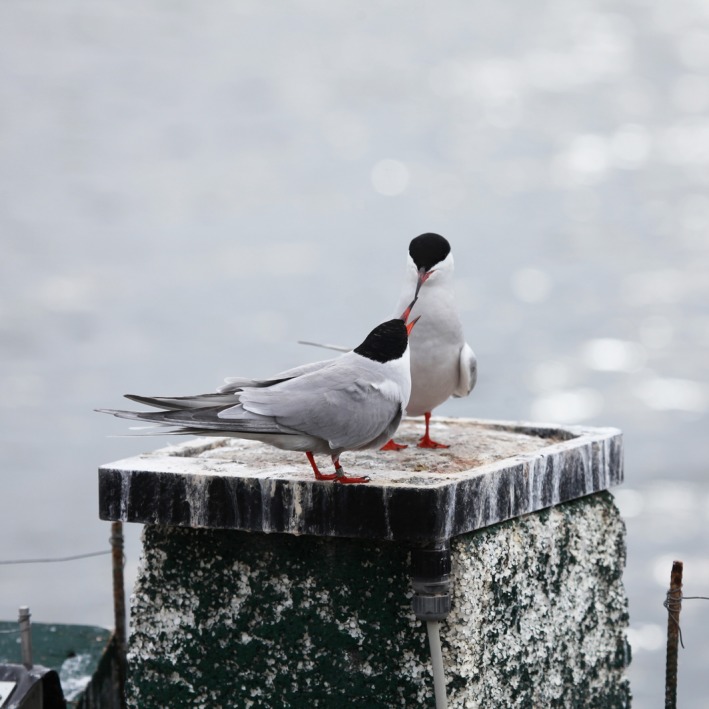
Two transponder‐tagged common terns using a resting platform equipped with an antenna for automated registration at the Banter See breeding colony (photo by S. Bouwhuis)

The response of seabirds to climate changes has received relatively little empirical attention, although consequences for reproductive success of mismatches between bird phenology and peaks in food abundance have been reported in zooplanktivorous seabirds (Hipfner, 2008). Our study therefore focused on the common tern (*Sterna hirundo*; Figure 1), a long‐lived colonial seabird and long‐distance migrant (Becker & Ludwigs, 2004) that should be particularly vulnerable to climate change (Both et al., 2010; Møller et al., 2008). In contrast to earlier breeding, which is the common response to climate changes of many bird species, common terns from our study population delayed egg laying between 1994 and 2006 (Ezard, Becker, & Coulson, 2007), even though early laying date was associated with improved annual reproductive success, as in most bird species (Arnold, Hatch, & Nisbet, 2004; Becker, 1996; Ezard et al., 2007). In the study colony of common terns, chick mortality before fledging increased over the study period, probably because of food shortage, which in combination with a decrease in subadult survival resulted in a population decline between 1984 and 2010 (Szostek & Becker, 2012).

In the context of a declining population and delay in the annual onset of reproduction, the aims of our study were (1) to confirm the delay in breeding with additional years of data; (2) to determine the evolutionary potential of the timing of reproduction through its genetic variance, its heritability, the direction and strength of natural selection acting on this trait, and potential constraints via genetic correlation with the timing of spring migration; and (3) to identify whether phenotypic plasticity could explain the delay in reproductive timing. As resource productivity and climatic conditions at the west‐central African wintering grounds may impact both migration and breeding ground population demographic events (Szostek & Becker, 2015; Szostek, Schaub, & Becker, 2014; Szostek et al., 2015), we also examined influences of climatic factors at the wintering grounds on reproductive timing.

## 2 Methods

### 2.1 Study site, data, and pedigree

Common terns were studied at Wilhelmshaven on the German coast along the North Sea. Between 1992 and 2010, the number of breeding pairs at the “Banter See” colony fluctuated between 90 and 530. Terns arrived from their wintering grounds (in subequatorial Africa; Becker et al., 2016) in April, laid the first eggs in early May, and returned to their wintering grounds in September (Becker et al., 2016). Terns nested on six artificial islands that are about 10.7 × 4.6 m and spaced 0.9 m apart. Nests were protected from flooding and rat predation by low concrete walls around the islands. From 1992 to 1995, an initial 101 adults were captured and marked with subcutaneous passive transponders, and since 1992, all fledglings have been marked with transponders (Becker & Wendeln, 1997).

From 1994, the arrival and presence of transponder‐marked terns was monitored via antennae on resting platforms (see Figure 1) that were affixed to the walls of the islands. Arrival date was defined as the first day of return to the breeding grounds after migration, and recorded as the number of days after 1 January for a given year. The automatic detection system (Becker, Ezard, Ludwigs, Sauer‐Guerth, & Wink, 2008) was extremely accurate, with a probability of resighting close to one (Szostek & Becker, 2012), due to high breeding site fidelity of common terns.

We restricted analyses to individuals that had prior breeding experience in at least 1 year (mean = 7.4 years old ± 2.9 *SE*, maximum age = 22 years, *N* = 2,787 clutches), because first‐time breeders, which are mostly three or 4 years of age (on average 3.5 ± 0.8 *SE*), exhibited a strongly different distribution of arrival and laying dates (Arnaud et al., 2013; Becker et al., 2008; González‐Solís, Becker, Jover, & Ruiz, 2004; Ludwigs & Becker, 2002) and extremely low reproductive success (Limmer & Becker, 2010). Surveys of the total colony every 2–3 days revealed when new clutches were initiated and new eggs were laid and nests and eggs were marked for subsequent recognition. At each nest, parents were identified using portable antennae placed around the nest for 1–2 days during incubation, which is shared by both partners. From these procedures, laying date of each marked pair was recorded as the number of days between 1 January and the inception of the laying event.

Chicks were ringed at hatching and checked every 2–3 days until they fledged (at about 26 days; Becker & Wink, 2003) or perished. We determined whether a clutch was the first or a replacement clutch (or a second clutch in rare cases) and for each clutch, the number of fledglings was recorded. Chicks were implanted with transponders (see above) prior to fledging. We built pedigrees from the records of parents and offspring. This social pedigree, which comprised 4,023 individuals, is a good approximation of the genetic pedigree, because both social parents were known and because breeding partners exhibit very low levels of extra‐pair paternity (1.3% of copulations and 2.9% of fertilizations were extra‐pair, González‐Solís, Sokolov, & Becker, 2001). The pedigree covered four generations, and the number of paternities and maternities was 2,377.

A given individual cannot start reproducing before arriving on the breeding grounds; hence, phenotypic measures of arrival and laying dates are statistically collinear. While we focused on breeding timing, we also examined the interval between arrival from migration and onset of breeding (i.e., the number of days between the arrival of the females from the spring migration and the start of the laying event) because it could be involved in the birds' phenological response to environmental conditions. We called this variable the “prelaying interval.” Analyses were thus based on multivariate models including both prelaying interval and laying date.

We ran linear models with year and age as continuous fixed effects on prelaying interval and laying date, in order to estimate the temporal changes in the variables between 1996 and 2010, while controlling for changes in the age structure of the population.

### 2.2 Animal models

In order to estimate additive genetic variances for laying date and the prelaying interval, as well as phenotypic and additive genetic correlations between these traits, we used restricted maximum‐likelihood mixed models (known as “animal models,” Kruuk, 2004) in a frequentist approach (ASReml software, Gilmour, Gogel, Cullis, & Thompson, 2009). We first performed a bivariate animal model with prelaying interval and laying date. Age was included as a fixed effect and year, individual identity, and additive genetic variation as random effects.

The total phenotypic variance ($\sigma_{p}^{2}$) of laying date or prelaying interval in experienced females was partitioned into the following components:

$$\sigma_{p}^{2}=\sigma_{\mathrm{pe}}^{2}+\sigma_{a}^{2}+\sigma_{r}^{2},$$

where $\sigma_{\mathrm{pe}}^{2}$ was the permanent environmental variance, that is, persistent influence of female identity over time with environmental origin, $\sigma_{a}^{2}$ the additive genetic variance, and $\sigma_{r}^{2}$ the residual variance.

To test whether variances were significantly different from zero, we used reduced models where the focal random effect was dropped. We compared the deviance from the reduced and complete models with a likelihood ratio test (LRT, Wilson et al., 2009). Tests of repeatability of arrival date, prelaying interval, and laying date followed the methods of Nakagawa and Schielzeth (2013).

### 2.3 Selection analyses

We measured the strength of selection presently acting on the two focal life‐history traits, using different individual fitness components: annual sum of fledglings *F*, survival of the focal individual to the next year *S*, and annual fitness *W*. The annual sum of fledglings corresponds to the sum of fledglings from the first clutch, the second clutch, and/or a replacement clutch during the focal year. The annual sum of fledglings was subjected to an augmentation of 1 to avoid null values. Following Qvarnström, Brommer, and Gustafsson (2006), annual fitness was calculated as:

W=S+F/2.

We obtained the relative annual sum of fledglings, relative survival, and relative annual fitness, by dividing each individual measure by the appropriate annual means (after McAdam & Boutin, 2003; Garant, Kruuk, McCleery, & Sheldon, 2007). Laying date and prelaying interval were standardized (zero mean, unit variance) within each year. First, we estimated (1) directional selection differentials (*s*_*t*_) using univariate linear models for each phenotypic trait (*t*, laying date or prelaying interval), followed by (2) quadratic selection differentials (${c^{2}}_{t}$) from models that included both a linear and a quadratic term (Lande & Arnold, 1983):

(1) $$w=\alpha +s_{t}x_{t}+\varepsilon ,$$

(2) $$w=\alpha +s_{t}^{\prime }x_{t}+1/2c_{t}^{2}x_{t}^{2}+\varepsilon ,$$

where *w* is the relative fitness, α is the intercept, and ε is an error term. Second, we estimated (3) standardized linear selection gradients for prelaying interval (β_PLI_) and for laying date (β_LD_) and (4) nonlinear quadratic gradients for prelaying interval (γ_PLI_) and for laying date (γ_LD_) including correlational (γ_PLI/LD_) selection gradients:

(3) $$w=\alpha +\beta_{\mathrm{PLI}}x_{\mathrm{PLI}}+\beta_{\mathrm{LD}}x_{\mathrm{LD}}+\varepsilon ,$$

(4) $$w=\alpha +\beta_{\mathrm{PLI}}x_{\mathrm{PLI}}+\beta_{\mathrm{LD}}x_{\mathrm{LD}}+1/2\gamma_{\mathrm{PLI}}{x_{\mathrm{PLI}}}^{2}+1/2\gamma_{\mathrm{LD}}{x_{\mathrm{LD}}}^{2}+\gamma_{\mathrm{PLI}/\mathrm{LD}}x_{\mathrm{PLI}}x_{\mathrm{LD}}+\varepsilon .$$

Quadratic coefficients and their standard errors were doubled, so that we could report them as stabilizing or disruptive selection gradients (Stinchcombe, Agrawal, Hohenlohe, Arnold, & Blows, 2008).

Statistical significance of the selection differentials and gradients were estimated using the raw data with generalized linear mixed models, respectively, with the identity of the individual as random effect and with Poisson error structure for fecundity and annual fitness and with binomial error structure for viability.

### 2.4 Genetic covariances between life‐history traits and fitness

Evolutionary change in a phenotypic trait under selection is best predicted by applying the Robertson–Price identity (Morrissey, Kruuk, & Wilson, 2010), which stipulates that the expected change in mean phenotypic trait between generations is equal to the additive genetic covariance between relative fitness and the trait. We therefore ran bivariate animal models in female experienced breeders with each combination of annual sum of fledgling and life‐history traits in order to estimate the additive genetic covariances and correlations between phenotypic traits and fitness.

### 2.5 Plasticity analyses

From light‐level geolocators, we know that common terns from the “Banter See” population spend their winters on the west‐central coast of Africa (Becker et al., 2016), although we do not know whether climatic factors in the wintering area or at stopovers along the migration route explain variation in the timing of migration and breeding (but see Szostek et al., 2015). Using sliding windows (e.g., Brommer, Rattiste, & Wilson, 2008; Husby et al., 2010; van de Pol & Cockburn, 2011) with an AIC selection process, we therefore explored the population response of breeding phenology to several global climatic indexes (Atlantic Multi‐decadal Oscillation, NAO, Southern Oscillation Index), as well as to average wind speed, sum of rainfall, and sea surface temperature (SST) at the nearest weather station (Cuxhaven, Germany). We did a similar analysis for SST recorded at the wintering grounds and points along the migration route (Senegal, Portugal, France, and the Netherlands). The sliding windows explored periods between August of the previous year and June of the focal year with a step of 1 month.

After the determination of the climatic variable with the strongest statistical relationship with laying date (i.e., SST in Senegal during the December month prior to breeding), we tested whether the laying date of females showed a plastic response to this climatic factor. This analysis included only experienced females with 2 or more years of breeding. We used a generalized mixed model with age as fixed effect, the identity of the female, and the interaction of female identity with SST in Senegal as random effects to quantify phenotypic plasticity. We additionally examined whether individual females exhibited plasticity in laying date by testing for variation in laying date both within and among individuals, using the techniques described by van de Pol and Wright (2009).

## 3 Results

**Figure 2:**
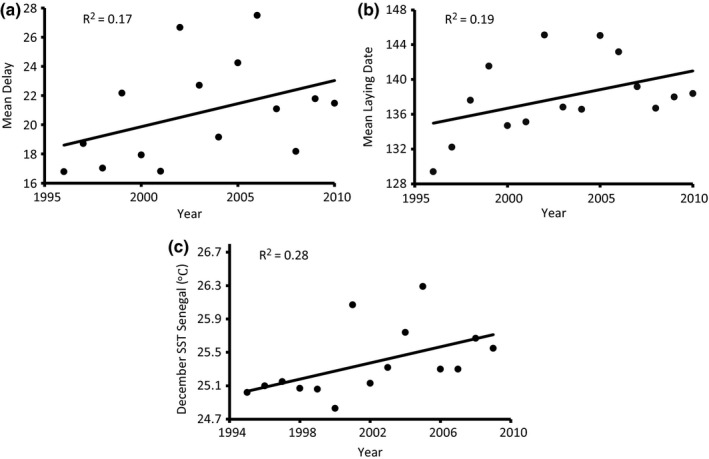
(a) Changes over time in the mean delay between arrival at the colony from migration and laying date for experienced breeding common terns. (b) Changes over time in mean laying date for experienced breeding common terns. (c) Changes over time in mean sea surface temperature in December in Senegal

Across the 15‐year study, the onset of reproduction was significantly delayed over time (β = 0.35 days/year ± 0.05 *SE*;* t* = 7.52; *p* < .0001; Figure 2b). This was due to a significant increase in the prelaying interval (β = 0.30 days/year ± 0.04 *SE*;* t* = 6.7; *p* < .0001; Figure 2a), because arrival dates of experienced breeders did not change significantly (β = 0.09 days/year ± 0.05 *SE*;* t* = 1.58; *p* = .12).

### 3.1 Evolutionary potential of phenology of breeding

Laying date was significantly repeatable, with about 32% of the variation (±3% *SE*) in laying date being accounted for by individual identity (Table 1). Laying date was also significantly heritable, with additive genetic variation accounting for about 27% (±9%) of the phenotypic variation. Prelaying interval was also significantly repeatable (18 ± 3%) but not significantly heritable (*h* ^2^ = 0.00 ± 0.05). A long prelaying interval was associated with a later laying event at the phenotypic level (*r* = .66 ± .03 *SE*,* t* = 25.7, *p* < .0001). Prelaying interval and laying date were not under significant survival selection (Table 2). In the univariate selection models; however, shorter prelaying intervals and early laying were associated with a higher annual sum of fledglings and greater annual fitness. In the bivariate selection models, only laying date was under significant negative directional selection via the annual sum of fledglings and annual fitness.

**Table 1:** Mean value, partition of the phenotypic variance, estimates of heritability and repeatability for prelaying interval (PLI), and laying date (LD) in female experienced common terns (±1 *SE*). Age, age^2^, and focal year were included as fixed effects

	*N*	*n*	Mean	$\sigma_{p}^{2}$	$\sigma_{r}^{2}$	$\sigma_{\mathrm{pe}}^{2}$	$\sigma_{a}^{2}$	*h* ^2^	*r* ^2^
PLI	1,269	273	23.7	52.09 ± 2.25	42.81 ± 1.92	9.28 ± 1.77	0.00 ± 3.88	0.00 ± 0.05	0.18 ± 0.03***
LD	1,352	277	139.7	67.03 ± 3.29	46.01 ± 2.00	2.77 ± 5.53	18.25 ± 6.38**	0.27 ± 0.09	0.32 ± 0.03***

*N*, total number of records; *n*, number of females; $\sigma_{p}^{2}$, phenotypic variance; $\sigma_{r}^{2}$, residual variance; $\sigma_{\mathrm{pe}}^{2}$, permanent environment variance; $\sigma_{a}^{2}$, additive genetic variance; *h* ^2^, narrow‐sense heritability; *r* ^2^, repeatability.

Significance of the additive genetic variance and repeatabilities: **p < .01; ***p < .0001.

**Table 2:** Univariate and bivariate selection analyses on standardized prelaying interval (PLI) and laying (LD) dates in common tern female experienced breeders (±1 *SE*). The results for all fitness components are presented: annual sum of fledglings (*F*), survival (*S*), and annual fitness (Ft)

	*F*	*S*	Ft
n	1,532	1,310	1,310
Standardized selection differentials			
s_PLI_	−0.051 ± 0.009***	−0.012 ± 0.009	0.045 ± 0.009**
${c^{2}}_{\mathrm{PLI}}$	−0.008 ± 0.010	−0.008 ± 0.008	−0.011 ± 0.009
s_LD_	−0.112 ± 0.009***	−0.017 ± 0.009	−0.093 ± 0.009***
${c^{2}}_{\mathrm{LD}}$	0.048 ± 0.009*	−0.019 ± 0.009	0.025 ± 0.010
Standardized selection gradients			
β_PLI_	0.025 ± 0.011	−0.003 ± 0.010	0.011 ± 0.011
γ_PLI_	−0.011 ± 0.011	−0.006 ± 0.010	−0.002 ± 0.011
β_LD_	−0.127 ± 0.011***	−0.015 ± 0.010	−0.100 ± 0.011**
γ_LD_	0.053 ± 0.011	−0.015 ± 0.011	0.026 ± 0.012
γ_PLI/LD_	0.012 ± 0.014	0.003 ± 0.013	0.005 ± 0.013

*s*, directional selection differentials; *c* ^2^, quadratic selection differentials; β, standardized linear selection gradients; γ, nonlinear quadratic gradients; γ_W/LD_, correlational selection gradient.

Significance: *p < .05; **p < .01; ***p < .0001.

Quantitative genetic analyses of the fitness components revealed nonsignificant additive genetic variances for survival and annual fitness ($\sigma_{a}^{2}<0.00001$, χ^2^ = 0, *p* = 1; and $\sigma_{a}^{2}=0.007\pm 0.009SE$, χ^2^ = 0.95, *p* = .33, respectively, *N* = 1,310), but significant additive genetic variance for the annual sum of fledglings in experienced females ($\sigma_{a}^{2}=0.027\pm 0.009\;SE$, χ^2^ = 5.5, *p* = .02, *N* = 1,310). Additive genetic variance for laying date in this sample was significant as well ($\sigma_{a}^{2}$ = 14.05 ± 6.01 *SE*, χ^2^ = 3.8, *p* = .05, *N* = 1,310). The genetic correlation between annual sum of fledglings and laying date in females was negative and approached significance (*r*_G_ = −0.65 ± 0.35, *p* = .06).

### 3.2 Climate results and phenotypic plasticity

**Figure 3:**
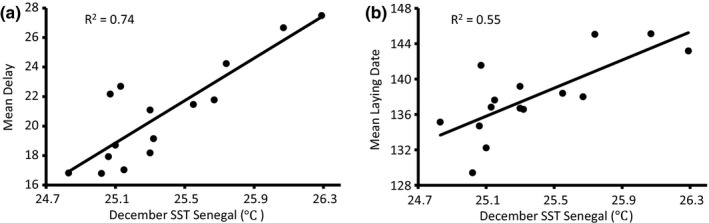
(a) The relationship between the mean delay between arrival at the colony from migration and laying date, and mean sea surface temperature (SST) in Senegal in December before the breeding season. (b) The relationship between mean laying date and mean SST in Senegal in December before the breeding season

At the population level, the sliding window approach showed that, among all the tested climatic factors, the strongest significant relationship was a positive relationship between both the delay from arrival to laying date and mean laying date itself, and mean SST in Senegal in the December prior to breeding (*R* ^2^ = .74, *n* = 15 years, *p* < .0001; *R* ^2^ = .55, *n* = 15 years, *p* < .002; Figure 3; see also Szostek & Becker, 2015; Szostek et al., 2015). Mean SST in Senegal in December showed a marked increase over the period 1995–2009 (corresponds to subsequent breeding in years 1996–2010; β = 0.05 ± 0.02 *SE*,* t* = 2.22, *p* = .045; Figure 2c).

For experienced common terns that bred in at least 3 years, we used a mixed model to examine the influence of SST in Senegal in December on laying date. Age and age‐squared were included in the model as fixed factors to control for the effects of aging (e.g., Zhang, Vedder, Becker, & Bouwhuis, 2015), and female identity and the interaction between female identity and SST were included as random variables. In this analysis, the interaction term represents the slopes of individual females for laying date regressed on values of December SST (i.e., to test for equality of reaction norm slopes among individuals). We then removed the interaction term and reran the model. A LRT did not reveal a significant difference between the models that included and excluded individual plasticity (i.e., the slopes of reaction norms were not significantly different), for female laying date in response to December SST in Senegal (change in *R* ^2^ = .12, number of observations = 1,263, number of females = 211; χ^2^ = 2.1, *p* = .15). For each increase of 1°C of SST temperature in December on the wintering grounds, laying date was delayed on average 6.7 ± 0.5 days (*t* = 13.31, *p* < .0001).

We further tested the pattern of plasticity using a partitioning of within and among individual effects of December SST on female laying dates, with female identity a random variable (van de Pol & Wright, 2009). In this analysis, both the within‐individual and among‐individual patterns were highly significant (number of observations = 1,263, number of females = 211; *t* = 5.9 and 9.3, respectively, both *p*s < .0001), but there was no significant interaction between these terms to suggest that females with a different average laying date differed in their level of plasticity. In support of this, a test of random slopes of individual females (*R* ^2^ ± 1.96 *SD* = 0.02 ± 0.03) indicated that less than 1% of the variation in the data could be explained by inclusion of the interaction term (LRT; χ^2^ = 4.7, *p* = .10). These analyses indicated that the slopes of the individual and population‐wide reaction norms were quite similar.

## 4 Discussion

### 4.1 Microevolution and changes in laying date

Spring timing of annual reproduction became significantly later for common terns, by about 5 days over 15 years. This delay did not appear to be due to later returns from annual migration to the wintering grounds, as there was no significant change in return dates (Arnaud et al., 2013; Ezard et al., 2007; Szostek et al., 2015). Rather, the change in reproductive timing appeared to primarily result from a significant increase in the time between arrival at the breeding grounds and the production of eggs. This increase might have been caused by changes in arrival condition that varied with marine food resources at the wintering grounds (Szostek & Becker, 2015; Szostek et al., 2015), although it might also have resulted from changes in local prey availability on the breeding grounds (see below). Either way, our objective was to evaluate the possible roles of microevolution and phenotypic plasticity in producing the temporal change in the timing of annual reproduction.

For laying date to be delayed via microevolutionary change, significant heritability and fitness differences in reproductive timing are required. As is commonly found in avian species (e.g., Sheldon, Kruuk, & Merilä, 2003; Brommer & Rattiste, 2008; and reviewed by Postma, 2014), laying date in common terns was strongly heritable, with over a quarter of the phenotypic variance in their timing of breeding being explained by additive genetic effects. Significant fecundity selection also occurred, but favoured *earlier* breeding, a result in agreement with patterns observed in other bird species (e.g., Brommer, Merilä, Sheldon, & Gustafsson, 2005; Charmantier et al., 2008; Gienapp, Postma, & Visser, 2006; Phillimore, Leech, Pearch‐Higgins, & Hadfield, 2016; Porlier et al., 2012; Reed et al., 2009; Sheldon et al., 2003; Teplitsky et al., 2011; Visser et al., 2015). This significant selection for earlier laying co‐occurred with a nearly significant (*p* = .06) and substantial negative genetic correlation between laying date and fecundity (*r*_G_ = −0.65 ± 0.35), which suggested that selection was not acting solely on the environmental component of the trait.

Based on the significant genetic variance, heritability, and selection acting at the genetic level, laying date would be predicted to display a low‐to‐moderate evolutionary potential to evolve in the direction of earlier breeding, unless the fitness variation associated with laying date was due to correlations between laying date and other measures of phenology, particularly arrival from migration at the breeding colony. Arrival has been suggested as a potential constraint for changes in timing of breeding in migratory birds, because of the necessary connection between the sequential events within the annual cycle and because of potential phenotypic and genetic correlations between the life‐history traits (Both & Visser, 2001; Coppack & Both, 2002; Teplitsky et al., 2011; but see Goodenough, Hart, & Elliot, 2011; Lourenco et al., 2011; Visser et al., 2015). Arrival date, however, was only marginally and weakly heritable and did not change significantly over the study period (see also Arnaud et al., 2013; Ezard et al., 2007; Szostek et al., 2015).

We therefore examined the prelaying interval, which became significantly longer during the study. We also found significant fecundity selection on prelaying interval in our univariate analyses, but its sign was negative (*i.e*., shorter intervals were selected, favouring earlier laying dates), and the prelaying interval exhibited virtually no heritability. It thus seems more likely that the apparent fecundity selection on prelaying interval was influenced by its correlation with laying date, rather than vice versa. All in all, the significant delay in reproductive timing, therefore, must have been primarily due to phenotypically plastic changes in laying date, which is the most general result found in bird studies (Charmantier & Gienapp, 2014).

### 4.2 Phenotypic plasticity and changes in laying date

Phenotypic plasticity is now recognized as a widespread response to climate changes in birds (e.g., Charmantier & Gienapp, 2014; Pulido, 2007b). Recent studies have shown that local temperature, and particularly an increase in temperature, can alter the timing of avian migration and reproduction (Phillimore et al., 2016; Schaper et al., 2012; Singh, Budki, Rani, & Kumar, 2012; Tottrup et al., 2010). Individual common terns also exhibited significant phenotypic plasticity in breeding phenology. Our search for environmental variables associated with this plasticity revealed a significant relationship between SST on the wintering grounds in Senegal during the previous December and individual changes in laying date. Over the study period, December SST in Senegal increased, and subsequent laying was delayed.

While the mechanism underlying this pattern is not well understood, it likely reflects poorer foraging conditions in warmer tropical waters (Szostek & Becker, 2015; Szostek et al., 2015), and thus, an increased need to replenish body condition before reproduction can occur at the breeding colony in the North Sea. Arrival mass, however, did not decline over the study period (Szostek et al., 2015). Alternatively, changes in SST might have coincided with changes in local resource conditions in Wilhelmshaven, with the latter actually causing the observed longer prelaying periods during the study. During courtship when males have to feed their female mates with high quality fish to produce eggs in a short period of time (González‐Solís et al., 2001; Nisbet, 1973; Wendeln, 1997), poor food availability occurred during the years 2002–2009 (Dänhardt & Becker, 2011) and might have delayed laying date via stronger competition for food resources and slow increases in adult body condition. Alternatively, aspects of body condition of the terns that must be replenished before laying and that are not associated with body mass may be involved. In this case, a role of SST in Senegal directly influencing delayed laying during the study would be supported. Further research should not only test these alternatives, but also examine the possibility of an association between SST in Senegal and local resource conditions in Wilhelmshaven during the prelaying period.

Sensitivity to environmental cues to adjust breeding timing displayed a significant genetic variance in the great tit *Parus major* (Nussey et al., 2005; Visser et al., 2011). We examined females that reproduced several times during their lifetimes and used the slope of laying date on December SST in Senegal to estimate reaction norms describing phenotypic plasticity relative to the environmental conditions. We found that both individuals and the population as a whole exhibited similar changes over time in laying date. Further, we found no evidence for significant variation in the slopes of the reaction norms among individual females, as indicated by the lack of significant influence of the interaction of female identity and December SST in Senegal on laying date, under two different statistical models that describe variations in phenotypic plasticity. Given our substantial sample of over 200 individual females, this suggests that the plastic responses of females to December SST in Senegal were fairly similar and that there thus was no evidence of a significant genotype by environment interaction. This suggests that strong selection for a change in plasticity is unlikely and that a substantial plastic response to wintering conditions can oppose natural selection and constrain adaptation.

## Conflict of Interest

None declared.
